# Effects of Sorafenib, a Tyrosin Kinase Inhibitor, on Adrenocortical Cancer

**DOI:** 10.3389/fendo.2021.667798

**Published:** 2021-05-24

**Authors:** Lidia Cerquetti, Barbara Bucci, Salvatore Raffa, Donatella Amendola, Roberta Maggio, Pina Lardo, Elisa Petrangeli, Maria Rosaria Torrisi, Vincenzo Toscano, Giuseppe Pugliese, Antonio Stigliano

**Affiliations:** ^1^ Endocrinology, Department of Clinical and Molecular Medicine, Sant’Andrea Hospital Rome, Sapienza University of Rome, Rome, Italy; ^2^ Clinic Pathology Unit, San Pietro Hospital Fatebenefratelli, Rome, Italy; ^3^ Department of Clinical and Molecular Medicine, Sant’Andrea Hospital Rome, Sapienza University of Rome, Rome, Italy; ^4^ Department of Molecular Medicine Rome, Sapienza University of Rome, Rome, Italy

**Keywords:** adrenal cancer, neoangiogenesis, sorafenib, apoptosis, intercellular junctions, spheroids, matrix metalloproteinase-9, epithelium-mesenchymal transition

## Abstract

The lack of an effective medical treatment for adrenocortical carcinoma (ACC) has prompted the search for better treatment protocols for ACC neoplasms. Sorafenib, a tyrosine kinase inhibitor has exhibited effectiveness in the treatment of different human tumors. Therefore, the aim of this study was to understand the mechanism through which sorafenib acts on ACC, especially since treatment with sorafenib alone is sometimes unable to induce a long-lasting antiproliferative effect in this tumor type. The effects of sorafenib were tested on the ACC cell line H295R by evaluating cell viability, apoptosis and VEGF receptor signaling which was assessed by analyzing VE-cadherin and β-catenin complex formation. We also tested sorafenib on an *in vitro* 3D cell culture model using the same cell line. Apoptosis was observed after sorafenib treatment, and coimmunoprecipitation data suggested that the drug prevents formation VEGFR-VE-cadherin and β-catenin proteins complex. These results were confirmed both by ultrastructural analysis and by a 3D model where we observed a disaggregation of spheres into single cells, which is a crucial event that represents the first step of metastasis. Our findings suggest that although sorafenib induces apoptotic cell death a small portion of cells survive the treatment and have characteristics of a malignancy. Based on our data we recommend against the use of sorafenib in patients with ACC.

## Introduction

Adrenocortical carcinoma (ACC) is a rare and malignant endocrine tumor with a worldwide incidence of approximately two cases per million people per year (1). The long term therapeutic results are limited and are largely dependent on tumor stage. Surgery is the treatment of choice for patients with primary and secondary tumors and for local recurrence (2).

Moreover several cytotoxic agents have been used as monotherapies or have been used in combination to treat advanced disease, and mitotane is the only available adrenal specific treatment for ACC; however, since it exerts a cytotoxic effect it has limited utility (3). Because of the lack of effective treatments for this cancer, efforts to improve medical protocols for ACC are continually sought out.

Sorafenib is an inhibitor of several receptor tyrosine kinases involved in the neoangiogenesis process, including Vascular-Endothelial Growth Factor Receptors-2 (VEGFR2) and 3 (VEGFR3), and Platelet-Derived Growth Factor (PDGF). In preclinical models, it has shown efficacy against a wide variety of tumors such as breast, colon, and pancreas carcinoma (4, 5). It has been shown to block tumor angiogenesis by inhibiting serine/threonine kinases and block cell proliferation by inducing apoptosis in different human tumor cell lines (6, 7).

Since sorafenib showed a broad spectrum of antitumor activity in preclinical studies (8–10), multiple clinical trials have been undertaken to further investigate the role of this drug alone or in combination with several chemotherapies for cancer treatment. For its antineoplastic abilities, sorafenib (Nexavar, BAY43-9006, Bayer Pharmaceuticals Corp., West Haven, CT and Onyx Pharmaceuticals Corp., Emeryville, CA) has been approved by the Food and Drug Administration (FDA) for the treatment of advanced kidney and hepatocellular cancer.

Since ACC shows high levels of vascular endothelial growth factor (VEGF) (1, 11), some studies have focused on assessing the activity of sorafenib both in preclinical tumor models and in patients with adrenocortical cancer.

Mariniello et al. (12) reported the effects of sorafenib and everolimus, a mTOR inhibitor used as an anti-cancer therapy, alone or in combination in the SW13 and H295R cell lines and in a xenograft ACC model respectively. The authors demonstrated that the drug combination produced marked synergistic growth inhibition, in comparison to single agent therapy, suggesting that simultaneous inhibition of several signaling pathways may be a more effective anticancer strategy than using a single agent (12). They observed a great apoptotic effect in SW13 and H295R cells after sorafenib treatment and a significant mass reduction with increased survival particularly in SW13 xenograft model undergoing the combined sorafenib and everolimus treatment (12). Based on these results the authors concluded by proposing that the combination of molecular targeted agents may have both antiangiogenic and direct antitumor effects, thus representing a new therapeutic tool for the treatment of ACC.

In contrast, the results of the phase II study, published by Berruti et al. (13), reported the effects of metronomic administration of chemotherapeutic paclitaxel and antiangiogenic sorafenib in patients affected by advanced ACC. They observed clear disease development with a dramatic tumor progression and a significant increase in neoplastic lesions that occurred at a much higher and faster rate than the months before the start of the trial, forcing them to suspend experimentation before the end of the study. The authors concluded that, despite the antiproliferative effects observed with sorafenib in the preclinical model, treatment in patients with advanced ACC should not be recommended.

Finally, O’Sullivan and colleagues (14), demonstrated limited sorafenib effectiveness in ACC patients. After exposure of the tyrosine kinase inhibitor, the patients did not have any objective response evaluation criteria in the solid tumors response. The authors conclude that future trials are needed that target other molecular pathways in ACC.

In the present study we aimed to understand the detailed mechanism of the cytotoxic effects of sorafenib that were observed both in preclinical and clinical studies related to ACC. For this purpose we evaluated the effect of sorafenib *in vitro* by using the H295R ACC cell line, which is a monolayer culture, and in a 3D cell culture model, that is intended to mimic the structure, activity and extracellular environment of an *in vivo* tumor (15).

## Materials and Methods

### Cell Culture and Treatments

The H295R (CRL-2128) cell line, was cultured to confluence in DMEM F-12 medium (Sigma-Aldrich, St.Louis, MO,USA). The medium was supplemented with transferrin (5 µg/ml; Sigma-Aldrich, Milan, Italy), sodium selenite (5 ng/ml; Sigma-Aldrich, Italy), L-glutamine (2.5 mM; Life-Technologies, Inc., Paisley, UK), and antibiotics (50 µg/ml streptomycin, 50 IU/ml penicillin) (Life-Technologies). H295R cells were mycoplasma free and were maintained at 37°C in a humidified atmosphere of 5% CO_2_ and 95% air. Cells were treated with sorafenib at a concentration of 5 µM which was chosen based on a dose response curve (data not shown).

### Trypan Blue Analysis

Cell number was determined using a hemocytometer, and viability was assessed by the ability to exclude trypan blue. After trypsinization, cells were suspended in phosphate-buffered saline (PBS) and mixed with an equal volume of 0.4% trypan blue in PBS and the percentage of stained cells was determined.

### Cell Cycle Analysis in Flow Cytometric Analysis

The cell cycle was studied by using bromodeoxyuridine incorporation (BrdU; Sigma-Aldrich, USA). Briefly, cells were pulsed with BrdU at a final concentration of 10 μM for 15 min. Pulse-labeling experiments were performed by adding 10 μM BrdU to the medium during the last 30 min before analysis. After 30 min, the cells were harvested, washed once in PBS, fixed in 70% ethanol and stored at 4°C before analysis. Samples were then incubated with a mouse monoclonal anti-BrdU antibody (Roche Diagnostics, Milan, Italy) in complete medium containing 20% FCS and 0.06% Tween 20 (Calbiochem, San Diego, CA, USA) at room temperature for 1 h. After washing in PBS, cells were incubated with FITC-conjugated rabbit anti-mouse IgG 1:20 (DAKO, Glostrup, Denmark) in PBS for 1 h. Finally, cells were stained with a solution containing 5 μg/mL PI and 75 KU/mL RNase in PBS for 3 h, the top line of the cytograms represent BrdU-positive cells.

### Quantification of Apoptosis by Flow Cytometry

Apoptosis induction was evaluated by terminal deoxynucleotidyl transferase-mediated dUTP nick end labeling (TUNEL) assay (Roche Diagnostics) by using flow cytometry (FCM). Briefly, trypsinised adherent cells and floating cells were pooled, washed once with PBS (Lonza, Basel, Switzerland) and fixed in 4% paraformaldehyde (Sigma-Aldrich, Italy) for 30 min. Samples were then permeabilized in 0.1% Triton X-100 (Sigma-Aldrich, Italy) and 0.1% sodium citrate (Sigma-Aldrich, Italy) and washed with PBS (Lonza). Each sample was incubated in a 50 μl reaction mixture (Terminal deoxynucleotidyl Transferase, TdT, and fluorescein-dUTP) for 1 h at 37°C, washed with PBS (Lonza) and then measured by FCM at 24, 48 and 72 h.

### Gelatin Zymography of Matrix Metalloproteinase-9

Levels of matrix metalloproteinase-9 (MMP-9) expression in H295R cells were analyzed by SDS-PAGE gelatin zymography as reported by Baragi VM et al. (16). Briefly, when cells were 80% confluent, they were treated with 5 µM sorafenib for 72 h, washed twice, trypsinized and subjected to electrophoresis under non-reducing conditions *via* 10% SDS-PAGE copolymerized with 1 mg/ml gelatin as a substrate. After the gel was washed with 2% Triton X-100 solution to remove SDS, it was incubated in activation buffer (50 mM Tris, 5 mM CaCl_2_, 0.5 uM ZnCl_2_, pH 7.4) for 24 h at 37°C. Gels were then stained with 0.05% Coomassie brilliant blue R-250 and destained in acetic acid. Not stained regions of the gel corresponding to the active MMP-2 and MMP-9 were quantified by densitometry using ImageJ analysis.

### Western Blotting Analysis

Cellular lysates were sonicated on ice, clarified by centrifugation at 20.000 g and stored at –80°C. An aliquot of the cell lysates was used to evaluate the protein content by colorimetric assay. A total of 50 μg of protein content was electrophoresed on a 10% polyacrylamide gel in the presence of SDS and then was transferred onto a nitrocellulose membrane. Blots were blocked for 1 h at room temperature with 5% nonfat dry milk in Tween-PBS buffer. Treated and untreated cells were incubated with the following antibodies: anti-vimentin 1:200 (Santa Cruz Biotechnology, CA, US) anti-MMP-9 1:200 (Santa Cruz Biotechnology, CA, US), anti-N cadherin 1:100 (Santa Cruz Biotechnology, CA, US), anti-Vinculin 1:4000 (Sigma Aldrich, MO, US). The visualization of the antigens was performed by enhanced chemiluminescent detection reagents by ECL. The analysis of bands was performed with ImageJ software (Image Processing and analysis in Java).

### Flow Cytometric Immunofluorescence

Cultured cells were harvested and the expression of cell surface markers was analysed by indirect immunofluorescence using a FACS Calibur cytofluorimeter (Becton Dickinson, Franklin Lakes, NJ, USA). For indirect immunofluorescence, cells were incubated with primary antibodies specific for N-Cadherin (1:100), VE-Cadherin (1:500), VEGFR2 (1:250), pVEGFR2 (1:500) and β-Catenin (1:500) (Santa Cruz Biotechnology, CA, US) for 1 h on ice and then incubated with a secondary FITC-conjugated antibody for 50 min on ice and immediately analyzed by FCM.

### Coimmunoprecipitation

H295R cell pellets were resuspended in a low stringency cell lysis solution (NP40 1%, leupeptin 1μg/ml, pepstatin 1μg/ml, aprotinin 2 μg/ml, phenylmethylsulphonylfluoride 0.2 mM, sodium fluoride 10 mM). Then, the samples were sonicated for 10 s (Branson sonifier 150, Carouge, Switzerland). Preclearing of the lysates was performed by adding protein A to the extracts and mixing for 1h at 4°C. After preclearing, the supernatant was again incubated with protein A and with VEGFR2 at 4°C overnight. Immunocomplexes were washed three times with the low stringency lysis solution and were resolved by SDS-PAGE. Following the transfer to membranes, proteins were detected both by a VE-Cadherin and a β-Catenin horseradish peroxidase-linked secondary antibody. The anti-VEGFR2 antibody was used to normalize the amount of immunocomplex for quantification. The visualization of the antigens was performed by enhanced chemiluminescent detection. The analysis of bands was performed with ImageJ software (Image Processing and analysis in Java).

### Conversion of H295R Into Spheres Cells

H295R cells were plated in non-adherent conditions: serum-free cell culture medium (Gibco, Gaithersburg, MD, USA), supplemented with 20 ng/ml epidermal growth factor (EGF) (Sigma-Aldrich, USA), 40 ng/ml bFGF (Sigma-Aldrich, USA) and B27 (Gibco), and 2 μg/ml heparin in 60 mm low-attachment culture dishes at a density of 1.9 × 10^6^ cells/dish. After four days of seeding, the cells formed primary floating sphere-like structures. These structures grew rapidly until day 7. At this time, before the obtained sphere-like structures became necrotic, we harvested them and resuspended them in Accutase enzymatic solution (Gibco) for five minutes at 37°C and then mechanically dissociated them into a single cell suspension. The cells were reseeded in the same non-adherent conditions as above, and secondary spheres were allowed to form.

### Morphometric Analysis of H295R Spheroids

For the three-dimensional (3D) morphological analysis of H295R spheroids, samples were examined under an Axiovert 200 inverted microscope (Zeiss, Oberkochen, Germany) equipped with differential interference contrast (DIC) optics. For quantitative image analysis, digital micrographs of at least 300 multicellular structures and single floating cells for each condition were randomly captured from three different experiments using an AxioCam MRm CCD camera (Zeiss). The projected area (A), perimeter (P) and two orthogonal diameters (a and b) were measured for each multicellular structure using Axiovision software (Zeiss). Sphericity, volume and size were subsequently calculated according to the previously proposed methods (17–19).

The sphericity of each structure was expressed by calculating the shape factor: $\Phi =\frac{\pi \sqrt{\frac{4A}{\pi }}}{P}$.

The volume (μm^3^) was corrected for shape factor (SFC) and calculated by applying: $V=\Phi \frac{4\pi }{3}{\left(\frac{P}{2\pi }\right)}^{3}$.

The size (μm) was determined by calculating the geometric mean diameter: $D_{G}=\sqrt{ab}$.

All 3D cellular structures were also categorized according to their morphology and classified as follows: tight spheroids (densely packed spheres with almost indiscernible individual cells), irregular aggregates (two or more cells organized in loose or compact aggregates that do not form the typical spheroid structure) and single floating cells.

### Transmission Electron Microscopy

H295R monolayer and spheroid cultures, treated or not as described above, were washed three times with PBS and fixed with 2% glutaraldehyde in PBS for 2 h at 4°C. Samples were postfixed with 1% osmium tetroxide in veronal acetate buffer (pH 7.4) for 1 h at 25°C, stained with uranyl acetate (5 mg/ml) for 1 h at 25°C, dehydrated in acetone and embedded in Epon 812 (EMbed 812, Electron Microscopy Science, Hatfield, PA, USA). Ultrathin sections, unstained or poststained with uranyl acetate and lead hydroxide, were examined under a Morgagni 268D transmission electron microscope (TEM) (FEI, Hillsboro, OR, USA) equipped with a Mega View II charge-coupled device camera (SIS, Soft Imaging System GmbH, Munster, Germany) and analyzed with AnalySIS software (SIS).

### Statistical Methods

To compare variables that do not assume a Gaussian distribution, a Mann-Whitney nonparametric test was used. The data are presented with the Tukey box-and-whisker plot, where the central box represents the interquartile ranges (IR; 25^th^ to 75^th^ percentile), the middle line represents the median, and the horizontal lines represent the minimum and the maximum value of observation range. The values are expressed as the median ± IR. To compare variables that assume a normal distribution, Student’s T tests were used. The values are expressed as the mean ± SE (standard error) from three independent experiments. A chi-square test was used to compare categorical variables. *P* values <0.05 were assumed to be statistically significant.

## Results

### Sorafenib Reduced Cancer Cell Proliferation

**Figure 1:**
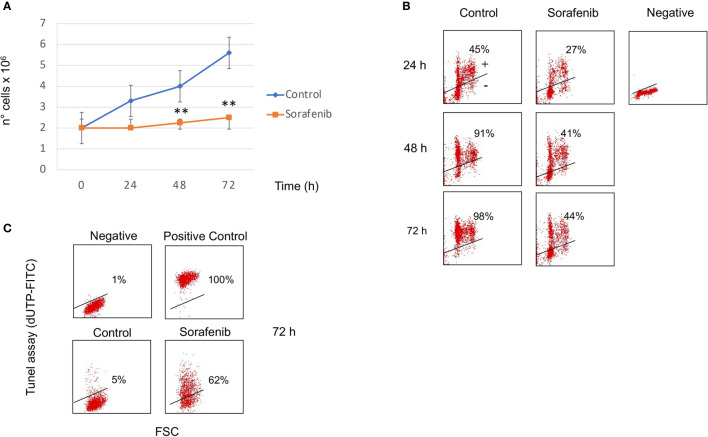
Sorafenib inhibits cell growth and cell proliferation. Viability curves of untreated and treated H295R cells with 5 µM sorafenib for 72 hours. Cell growth inhibition progressively increases and, at the highest concentration at 72 h, reaches 42% of inhibition after sorafenib treatment. The results represent the mean ± s.d. of three independent experiments done in duplicate. A comparison of the individual treatment was conducted by using ANOVA followed by the Tukey–Kramer *post hoc* test. **P <0.01 vs Ctrl **(A)**. Analysis of BrdU incorporation in control and in treated cells for 24h, 48h and 72 hours respectively. In samples treated with sorafenib about 40% BrdU incorporation was evident at 48-72 h of treatment. Representative results are shown, and were quantified from three independent experiments; each group was analyzed in duplicate **(B)**. TUNEL assay to evaluate induction of apoptotic cell death in H295R cells by sorafenib treatment. Flow cytometric analysis of untreated and treated cells with sorafenib for 72 h. At this time 62% of treated cells were dUTP-FITCH positive revealing apoptotic cell death. Similar results were obtained in three independent experiments **(C)**.

To assess the effects of sorafenib on cancer cell proliferation, H295R cells were treated with 5 µM of sorafenib for 72 h. As shown in **Figure 1A** the drug exposure showed a 24% inhibition of cell growth at 48 h, increasing 42% after 72 h compared to untreated cells (p<0.01). Cell viability in treated and untreated cells was assessed by trypan blue exclusion test to determine if cell viability was maintained after sorafenib treatment. We observed an integrity of the cell membrane after drug exposure, infact no alteration of cellular viability was observed during sorafenib treatment (data not shown), meaning that sorafenib did not induce toxicity.

BrdU incorporation was performed to determine if sorafenib treatment affected the cell cycle as well. **Figure 1B** shows that about 40% BrdU incorporation was evident at 48-72 h of treatment while in untreated cells incorporation was 91% and 98% after 48 h and 72 h respectively. These results suggest that the sorafenib used in this study successfully inhibited the growth of H295R cells according to a previous study (12).

### Sorafenib Induced Apoptosis in H295R Cells

To evaluate whether cell growth inhibition was attributed to apoptotic death we performed the TUNEL assay analyzed by FCM analysis. As evidenced in **Figure 1C**, sorafenib was able to induce apoptotic cell death (62% vs untreated cells) which was consistent with data from Mariniello and colleagues (12). They estimated an apoptotic percentage of 43% after sorafenib treatment in the same cell model.

### Sorafenib Inhibited Cell Proliferation and Did Not Affect VEGFR2 Expression

**Figure 2:**
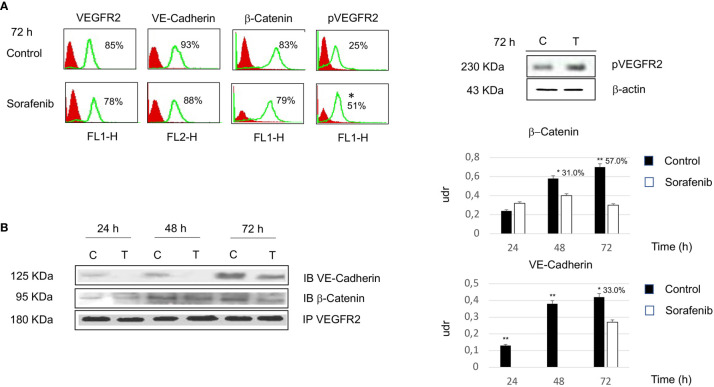
Flow cytometry analysis of VEGFR2, VE-cadherin, β-catenin and pVEGFR2 in untreated and sorafenib treated H295R cells. Only pVEGFR2 was significant in sorafenib treated sample (p<0.05). Western blot analysis of pVEGFR2 at 72 h is shown on the side **(A)**. Coimmunoprecipitations of VE-Cadherin and β-Catenin with VEGFR2. On the right, graph bars corresponding to densitometric analysis of β-Catenin and VE-Cadherin. Next to the bar graphs are reported the percentage of coprecipitate proteins at 24, 48 and 72 h. The samples treated with sorafenib and marked on top of the bar are considered statistically significant (*p<0.05;**p<0.01) **(B)**. The values of flow cytometry analysis and coimmunoprecipitations are expressed as the mean ± SE (standard error) from three independent experiments.

To investigate whether the effects of sorafenib on cell growth inhibition were correlated with the modulation of VEGFR2 protein, we assessed protein expression by immunofluorescence. The results obtained, revealed that the expression of the VEGFR2 protein did not significantly change after 72 h of sorafenib treatment. On the contrary the drug was able to increase p-VEGFR2 after the same time (approximately 50% vs untreated cells) (p<0.05) (**Figures 2A** and **5A**).

Then, we investigated whether VE-cadherin and β-catenin, whose action is mediated by the establishment of cadherin-based junctions, could be involved in the anti-proliferative and anti-angiogenic effects of sorafenib (20). As highlighted in **Figure 2A** the expression level of both VE-cadherin and β-catenin is similar to the levels observed for each in untreated cells. These results demonstrate that the effects of sorafenib on cell proliferation did not interfere with the expression levels of VEGFR2, VE-cadherin and β-catenin.

### Sorafenib Interfered With Intercellular Junctions

Although we did not observe a change in VEGFR2, VE-cadherin and β-catenin protein expression following sorafenib treatment, we wanted to test whether the drug interfered with the formation of the protein complex, which is involved in the proliferative and angiogenetic processes. Thus coimmunoprecipitation experiments were performed using H295R cells to determine the effects of sorafenib on intercellular junctions.

As shown in **Figure 2B**, VE-cadherin did not coprecipitate with VEGFR2 at 24 and 48 h. Moreover, we observed that 33% of the protein was in complex after 72 h of sorafenib treatment (p<0.05). In contrast changes in protein-protein interactions after sorafenib treatment were evidenced for β-catenin, in fact a 31% and 57% of the protein was in complex with VEGFR2 at 48 h (p<0.05) and 72 h (p<0.01) respectively. These data could indicate that sorafenib destabilizes the protein-protein interactions of a complex that is implicated in intercellular junctions.

### Sorafenib Affected the Ability of H295R Cultures to Grow as Tight Spheroids

**Figure 3:**
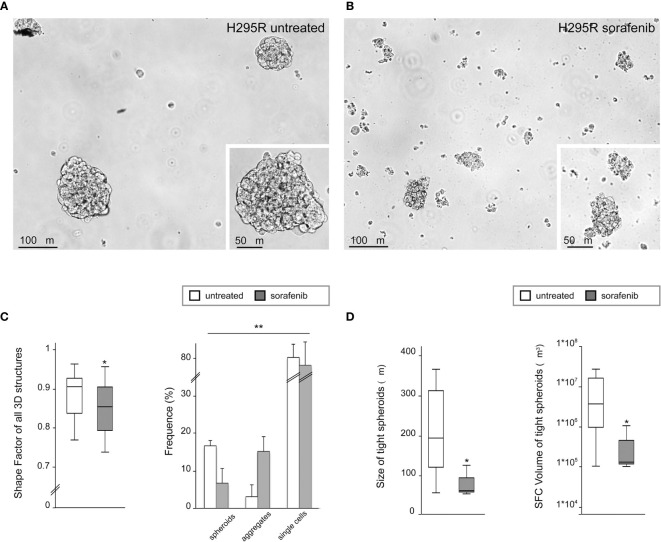
Three-dimensional and morphometric analysis of H295R spheroid growth in sphere medium with or without sorafenib. Differential interference contrast microscopy of H295R cell cultures: typical spheroid pattern of growth in untreated H295R cultures **(A)** and multicellular aggregates in H295R cultures cell growth treated with sorafenib **(B)**. Morphometric analysis of all multicellular structures from treated or untreated H295R cells: the box-and-whisker plot of shape factor shows a higher sphericity index for the spheroids derived from H295R untreated cultures (control) than for those from the treated cultures. The central box represents the interquartile ranges, the middle line represents the median and the horizontal lines represent the minimum and the maximum value of the observation range (Mann-Whitney test: *p<0.001). The results reported in the graph represent the mean values ± standard error (chi-squared test: **p<0.01) **(C)**. Morphometric analysis of tight spheroids from treated or untreated H295R cells: the box-and-whisker plots of size and SFC volume show a significant difference in untreated and treated cultures H295R cells (Mann-Whitney test: *p<0.001) **(D)**.

To analyze the 3D morphology and assess the morphometric parameters of H295R spheroids grown in sphere medium with or without sorafenib, DIC microscopy and digital image analysis techniques were performed as described in the *Materials and Methods*. Untreated H295R cultures (control) displayed a typical spheroid pattern of growth with densely packed spheres; in contrast, the H295R cell growth in the presence of sorafenib was characterized by a higher number of irregular multicellular aggregates (**Figures 3A, B**). In fact, the sphericity index of multicellular structures was significantly higher in untreated cultures with a shape factor of φ = 0.90 vs 0.85 in treated H295R cultures cells; also the percentage of tight spheroids was higher in untreated cultures (16.7% vs 6.7%; p<0.001) with fewer irregular aggregate compared with those of H295R cultures cells treated with sorafenib (3.1% vs 15.2%; *p*<0.01) (**Figure 3C** and **Table 1**).

**Table 1:** Morphological characterization and morphometric analysis of three-dimensional multicellular structures obtained from H295R cells cultured in sphere medium with or without sorafenib.

	All 3D structures	Tight spheroids				Irregular aggregates				Single floating cells
	Shape Factor^†^ (IR)	Frequency (% ± SE)	Shape Factor^†^ (IR)	Volume^‡^ (IR)	Size° (IR)	Frequency (%± SE)	Shape Factor^†^ (IR)	Volume^‡^ (IR)	Size° (IR)	Frequency (% ± SE)
**H295R untreated**	0.90 Φ	16.7 ± 3.8	0.93 Φ	3.7x10^6^ μm^3^ (0.9–22.7x10^6^)	194 μm	3.1 ± 3.9	0.83 Φ	7.2x10^5^ μm^3^ (0.3– 7.4x10^6^)	111.5 μm	80.2 ± 6.2
	(0.83–0.92)		(0.91–0.95)		(121–312)		(0.79–0.89)		(80–236.8)	
**H295R sorafenib treated**	0.85 Φ	6.7 ± 1.4^**^	0.91	1.3x10^5^ μm^3^ (1.1–4.5x10^5^)^*^	63 μm	15.2 ± 3.1^**^	0.81 Φ	2.3x10^5^ μm^3^ (1.3– 5.2x10^5^)^*^	76.5 μm	78.1 ± 3.5^**^
	(0.79–0.90)^*^		(0.90–0.93)^^^		(59–95)^*^		(0.77–0.86)^**^		(65–100.5)^^^	

^†^Shape Factor for spherical shape = 1; ^‡^Volume: parameter based on project area and correct for Shape Factor Φ; °Size: parameter based on geometric mean diameter of the multicellular structures (see Materials and Methods).

Statistics (vs H295R untreated):

^p=Not Significant (Mann–Whitney test);*p<0.001 (Mann-Whitney test); **p<0.01 (chi-square test). The sphericity index of multicellular structures was significantly higher in untreated vs treated cultures (p<0.01); the percentage of tight spheroids was higher in untreated cultures (p<0.001) with fewer irregular aggregate compared with those of H295R cultures cells treated with sorafenib (p<0.01). Size and SFC volume in untreated cultures showed a higher size and volume of tight spheroids compared to treated H295R cells (p<0.001).

Morphometric analysis of tight spheroids revealed that size and SFC volume in untreated cultures were characterized by a higher size and volume of tight spheroids compared to what was observed in the treated H295R cells (p<0.001) (**Figure 3D** and **Table 1**).

### Sorafenib Induced Cellular Damage in H295R Cells

**Figure 4:**
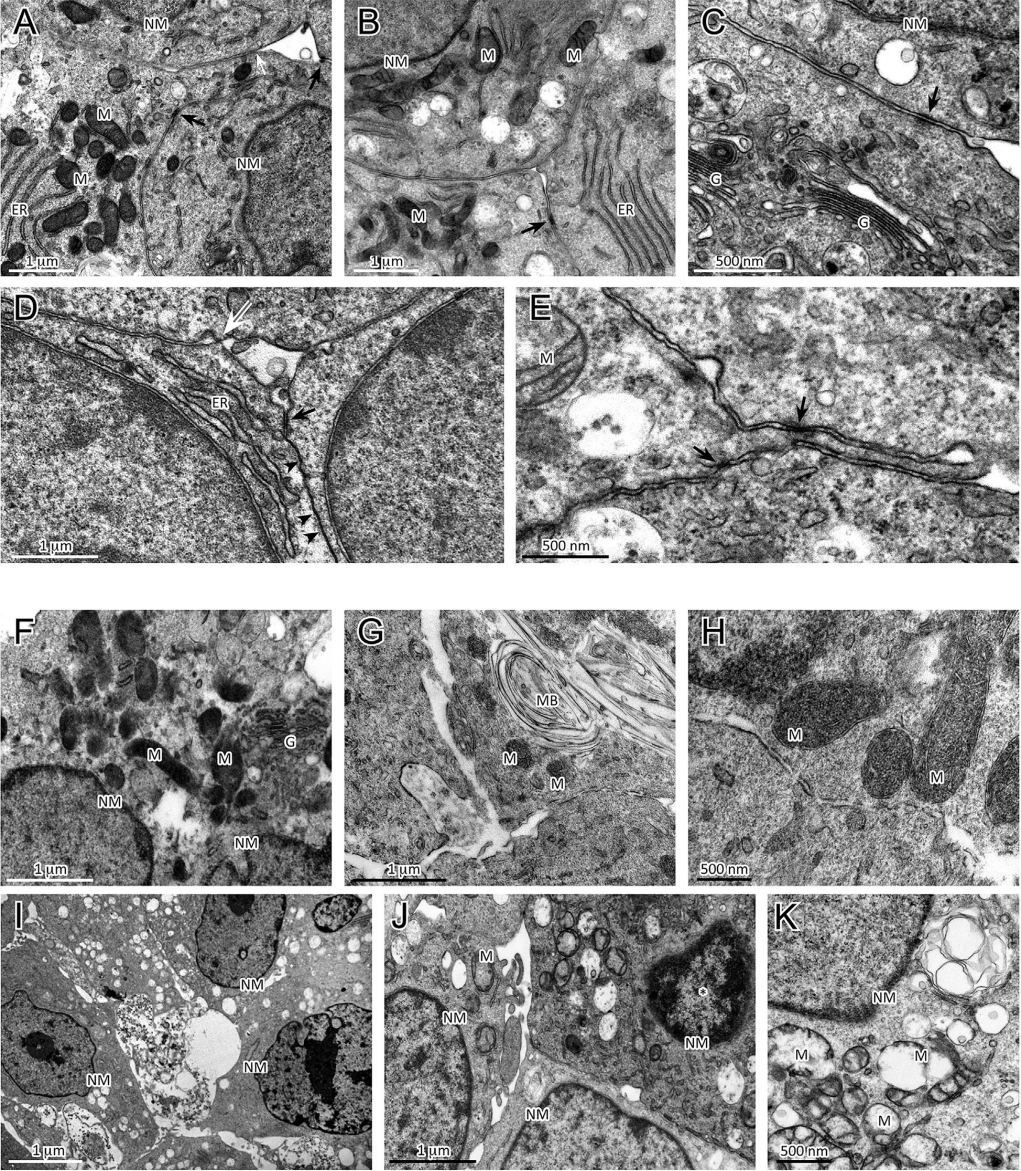
Analysis of ultrastructural features of cellular damage induced by sorafenib 5 µM at 72 h in H295R cultures. The H295R untreated monolayers **(A–C)** and spheroids **(D, E)** show nuclei with finely dispersed chromatin and prominent nucleoli, and cytoplasms with numerous mitochondria, a variable number of organelles, a prominent Golgi apparatus, many vesicles and a well-developed rough endoplasmic reticulum. Areas similar to tight junctions (white arrow), intermediate junctions (black arrows) and some tight junction-like regions (black arrowheads) are visible. Sorafenib treated H295R monolayers **(F–H)** and spheroid **(I–K)** cultures displayed apoptotic nuclei with areas of marginal and dense stained chromatin (**J**, asterisk) and nuclear and cellular fragmentation. The mitochondria are swollen and exhibit a loss of internal cristae. Cytoplasmatic vacuolization, swelling of rough endoplasmic reticulum cisternae and myelinic bodies were observed. H295R-treated spheroids show only a few intermediate-like junctions. Legend: NM, Nuclear membrane, M, Mitochondrion; ER, Endoplasmic reticulum; G, Golgi complex; MB, Myelinic body (considered as histological artifact).

To further characterize the morphological changes related to the sorafenib 5 µM treatment, the ultrastructural features of H295R monolayer and spheroid cultures were analyzed by TEM analysis at 72 h. The untreated H295R monolayers (**Figures 4A–C**) and spheroids (**Figures 4D, E**) had nuclei that were rounded or occasionally lobulated, and finely dispersed chromatin and prominent nucleoli. The cytoplasm displayed numerous rod-shaped or elongated mitochondria, variable amounts of organelles, a prominent Golgi apparatus, many cytoplasmic vesicles and a well-developed rough endoplasmic reticulum. Areas similar to tight junctions, as well as intermediate junctions and some tight junction-like regions were visible.

In contrast, the sorafenib-treated H295R monolayers (**Figures 4F–H**) and spheroids (**Figures 4I–K**) exhibited several ultrastructural characteristics of cellular damage. Apoptotic nuclei with areas of marginal, dense stained chromatin and nuclear fragmentation were visible. Most of the mitochondrial structures appeared swollen with a subtotal loss of internal cristae, while large vacuoles, swollen cisternae of endoplasmic reticulum and myelinic bodies (histological artefact) were also noticeable. Treated H295R spheroids did not exhibit classical junctional complexes and even exhibited few intermediate-like junctions.

### Sorafenib Treatment Promoted Tumor Progression and Invasiveness in the H295R Cell Line

**Figure 5:**
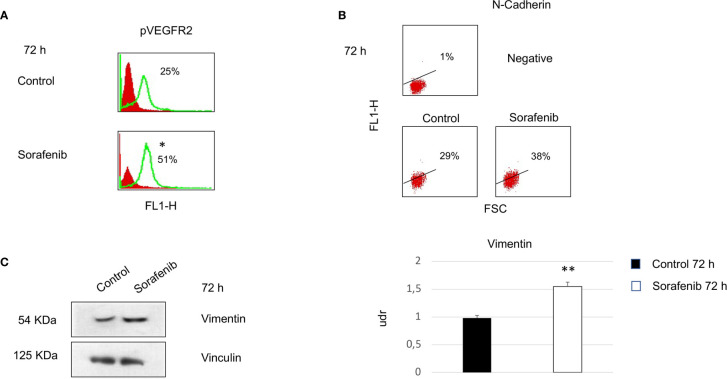
Cells were analyzed at 72 h after sorafenib treatment. Indirect immunofluorescence was analyzed by flow cytometric histograms for pVEGFR2 (particular of **Figure 2A**) (*p<0.05) **(A)** and by dot plot for N-cadherin in sorafenib-treated and untreated cells **(B)**. Western blot analyses of Vimentin and Vinculin were performed with 50 µg of protein from untreated and treated cells (**p<0.01) **(C)**. Proteins were resolved by 10% SDS-PAGE to anable analyses with the anti-vimentin or anti-vinculin antibodies.

**Figure 6:**
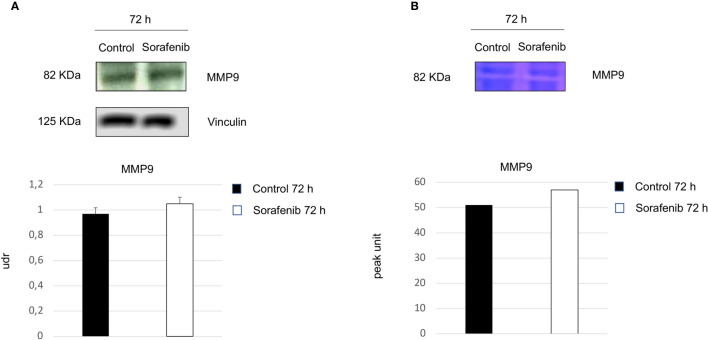
Role of sorafenib treatment in tumor invasiveness. MMP9 protein levels were detected by Western blot **(A)**. Zymographyc analysis confirmed the protein activity **(B)**. Below are shown bar graphs expressing the protein expression of MMP9 (on the left) and MMP9 activity (on the right). The results reported in the graph represent the mean values ± standard error. Densitometry summarizes the results obtained in three independent experiments.

Surprisingly, our study demonstrated an increase in p-VEGFR2 after 72 h of sorafenib treatment (approximately 50% vs untreated cells) (**Figures 2A** and **5A**). These results prompted us to verify whether sorafenib could paradoxically promote tumor progression. Thus, we determined the expression level of N-cadherin and vimentin, which are prognostic markers of tumor progression. As evidenced in **Figure 5B** the N-cadherin expression level, measured by immunofluorescence staining, showed an upregulation of approximately 10% compared to untreated cells. A similar increase was also observed for the level of vimentin (10% vs untreated cells) analyzed by Western blot (**Figure 5C**). Both of these results were observed after 72 h of sorafenib treatment. Finally, to evaluate the involvement of drug treatment on tumor invasiveness, we performed Western blot analyses of MMP-9 protein. As shown in **Figure 6A** sorafenib treatment induced an increase in the protein level of approximately 10% over the levels observed in the untreated cells. A similar result was obtained by zymographyc analysis, confirming up-regulation of the protein expression level of by approximately 10% (**Figure 6B**). All these data demonstrated that sorafenib failed to have an antiproliferative effect on a small population of tumor cells with tumor aggressiveness features.

## Discussion

Sorafenib is an inhibitor of several receptor tyrosine kinases involved in neovascularization, including VEGFR2, VEGFR3, and platelet-derived growth factor (5); it has shown efficacy against a wide variety of tumors in preclinical models, such as breast, colon, and pancreas carcinoma, and it has been approved for the treatment of hepatocellular carcinoma (9, 21). The mechanism of antineoplastic action of sorafenib lies primarily in its induction of apoptosis (7). Since sorafenib showed a broad spectrum of antitumor activity in preclinical studies (10–12), multiple clinical trials have been undertaken to further investigate its role, either alone or in combination with different chemotherapies for the treatment of several neoplasms (4, 6, 8, 10). Because of its activities, sorafenib has been approved by the FDA for the treatment of patients with advanced renal cell carcinoma, unresectable hepatocellular carcinoma and locally recurrent or metastatic, progressive differentiated thyroid carcinoma refractory to radioactive iodine treatment.

Despite the antineoplastic effects described for sorafenib, some patients may exhibit neoplastic progression during therapy with this drug as demonstrated by the comparison of the progression-free survival curves between treated patients and controls (22, 23).

Finally, numerous animal studies have also suggested that antiangiogenesis drugs may, in certain situations, actually accelerate metastatic spread which is recognized as a new form of adaptive resistance used by cancer cells (23, 24).

ACC is a highly vascularized neoplasia (1, 11) and metronomic chemotherapy is thought to be effective against ACC mainly by inhibiting tumor angiogenesis. Moreover, the antiangiogenic activity of metronomic chemotherapy can be theoretically increased by the concomitant administration of an antiangiogenetic drug (25). Therefore, there is a strong rationale for testing the combination of a metronomic chemotherapy with antiangiogenetic drugs in the management of ACC.

Berruti et al. tested the combination of daily sorafenib and weekly paclitaxel therapy in patients with progressive metastatic ACC in progression following treatment with mitotane in combination with one or two chemotherapy lines (13); the test was carried out as a multicenter, prospective phase II trial. The results of this study documented a progressive disease in nine consecutive patients at the first restaging dose after 2 months, which led to the interruption of the clinical trial. Furthermore in many patients, the tumor progression was dramatic, and the increase in the size of the tumor lesions was faster than it was in the months before the trial. These data suggest that this combination therapy may have paradoxically favored tumor progression. Although the data regarding the progression of tumors during treatment with sorafenib can be found in the literature (22–24) there is little data available regarding the mechanism by which sorafenib can elicit a malignant phenotype. In this regard the primary objective of our study was to determine whether sorafenib was able to induce a malignant phenotype in ACC *in vitro*.

For this purpose we conducted experiments in both 2D monolayer and 3D models. We chose a new approach, *in vitro* 3D cultures, since it represents an additional step that can bridge the gap between conventional 2D monolayer cultures and animal models. Additionally, this approach is especially useful for studying the invasive proprieties and metastatic potential of tumor cells, thus facilitating the development and screening of new drugs (17, 18, 26).

The results obtained from our study demonstrated that sorafenib induces cell growth inhibition due to a significant increase in apoptosis. The treatment also caused a destabilization of intercellular junctions by altering the formation of the protein scaffold; this alteration was revealed by coimmunoprecipitation experiments, in which an absence of the complex formation between VE-cadherin and β-catenin was evident after sorafenib exposure. Furthermore, we used ultrastructure analysis to demonstrate the disaggregation of the spheres into single cells after sorafenib treatment.

However, following treatment, we noticed an increase in the amount of the phosphorylated form of VEGFR2. This result contrasts with a well-known effects of sorafenib, which is interference with the angiogenetic process, leading to a reduction in VEGFR expression. These steps are crucial for tumor growth, progression, and metastasis. These apparently contradictory results have been observed by other authors and support both the resistance to sorafenib that occurs in some cancers and the lack of long-term response to sorafenib treatment (27–29).

This prompted us to investigate the molecular processes and the angiogenetic factors associated with tumor progression.

Tumor spheroid cell culture models have been used to study the responses of ACC to sorafenib treatment. H295R cells have the capacity to form spheroids, as reported by Lichtenauer and colleagues (30). The ability to form spheroid colonies is a recognized method used to identify cancer stem cells displaying an enhanced tumorigenic ability (31). This specific cell population is thought to be closely linked to the epithelium-mesenchymal transition (EMT), which is defined as crucial in metastatic spread and in tumor recurrence (32, 33). In cancer, EMT is associated with poor survival for the patients and seems to be a key step in the development of metastasis (32), since cells lose their polarity and cell-to-cell contacts and therefore become more motile (33, 34).

Evidence that exposure to sorafenib leads to EMT in ACC neoplasms come from analyzing some of the markers involved in this transition, such as N-cadherin and vimentin. Our results showed an increased expression level of both of these proteins. We noticed that only a small percentage of cells (approximately 10%) revealed disregulation of this protein as revealed by FACS analysis and by densitometric analysis of the proteins.

Furthermore, in our study, we observed that although sorafenib induced apoptosis, a small percentage of cells appeared to be resistant to treatment and exhibited the features of invasive phenotype, as evidenced by increased levels of MMP-9 after sorafenib treatment. MMP-9 is recognized as an enhancer protein in the metastatic process. Therefore it is clear that this population of cells is resistant to treatment and shows features of invasiveness and malignancy. Our results were in agreement with those described by van Malenstein et al. (35) who reported a direct effect of sorafenib on the epithelial cells by inducing a malignant phenotype. It was also demonstrated that sorafenib targets cofilin, which negatively regulates the polymerization of actin to disrupt the cytoskeleton, and cause cells detachment (36). Our results, which are in accordance with previous studies performed and are confirmed by Berruti’s clinical trial could explain why sorafenib treatment elicits a malignant phenotype, in patients with ACC to cause poor and devastating results. Based on these data, we warn against the clinical use of sorafenib as a therapeutic strategy for the treatment of ACC.

## Data Availability Statement

The original contributions presented in the study are included in the article/supplementary material. Further inquiries can be directed to the corresponding author.

## Author Contributions

LC and AS conceived the original idea. LC, BB, DA, SR, PL, and RM carried out the experiments. LC, BB, and AS wrote the manuscript with support from SR. EP, MT, and AS supervised the project. VT and GP performed data curation. AS funding acquisition. All authors contributed to the article and approved the submission version.

## Funding

This work was supported by Sapienza University of Rome “Progetti d’Ateneo”: grant No. RM1181643692671C (to AS).

## Conflict of Interest

The authors declare that the research was conducted in the absence of any commercial or financial relationships that could be construed as a potential conflict of interest.
